# Associations of Anger Expression and Social Anxiety with Cortisol, the Cortisol/DHEA-S Ratio, and Interleukin-6 in Psychiatric Outpatients: An Exploratory Cross-Sectional Study

**DOI:** 10.3390/brainsci16101134

**Published:** 2026-10-20

**Authors:** Junho Choi, Chun Il Park, Shin Tae Kim, Jee In Kang, Se Joo Kim, June-ho Seo

**Affiliations:** 1 Department of Psychiatry, Yonsei University Wonju Severance Christian Hospital, Wonju 26426, Republic of Korea; cjunho2@gmail.com; 2 Department of Psychiatry, Yonsei University College of Medicine, Seoul 03722, Republic of Korea; chunil1001@yuhs.ac (C.I.P.); skim238@yuhs.ac (S.T.K.); jeeinkang@yuhs.ac (J.I.K.); kimsejoo@yuhs.ac (S.J.K.); 3 Institute of Behavioral Science in Medicine, Yonsei University College of Medicine, Seoul 03722, Republic of Korea; 4 Department of Psychiatry, Yonsei University Wonju College of Medicine, Wonju 26426, Republic of Korea

**Keywords:** cortisol, dehydroepiandrosterone sulfate, cortisol/DHEA-S ratio, interleukin-6, hypothalamic–pituitary–adrenal axis, anger expression, social anxiety

## Abstract

**Highlights:**

**What are the main findings?**

- Social anxiety was associated with higher IL-6 after FDR correction and remained associated after accounting for anger-in in a separate analysis.
- Anger-out showed inverse associations with morning cortisol and the cortisol/DHEA-S ratio, but neither remained significant after FDR correction.

**What are the implications of the main findings?**

- Joint assessment of anger suppression and social anxiety may help clarify their biological correlates.
- The findings support symptom-focused psychiatric biomarker research and warrant longitudinal replication.

**Abstract:**

**Background:** Chronic psychological stress can alter hypothalamic–pituitary–adrenal (HPA) axis activity and inflammatory signaling. However, the biological correlates of specific stress-related emotional experiences and expression patterns remain incompletely characterized in clinical populations. We therefore examined the associations of anger expression and social anxiety with HPA-axis-related and inflammatory biomarkers in psychiatric outpatients. **Methods:** In this exploratory, single-center cross-sectional study, clinical records of 85 adults attending their initial visit to the study clinic were retrospectively reviewed. Anger expression was assessed with the Korean State-Trait Anger Expression Inventory (STAXI-K; n = 61) and social anxiety with the Korean version of the Liebowitz Social Anxiety Scale (LSAS; n = 63). Fasting early morning serum concentrations of cortisol, dehydroepiandrosterone sulfate (DHEA-S) and interleukin-6 (IL-6) were measured. Hierarchical linear regression and partial correlation analyses were conducted adjusting for age, sex and body mass index. Additional analyses included left-censored Tobit models and cross-equation Wald tests. **Results:** Higher social anxiety scores were associated with higher log-transformed IL-6 (n = 63; β = 0.400, *p* = 0.001), which was the only association that remained significant after false discovery rate (FDR) correction across the nine main predictor–biomarker tests (q = 0.013). This association also remained significant in the reported sensitivity analyses. Higher anger-out scores were associated with lower morning cortisol (n = 61; β = −0.311, *p* = 0.022) and a lower log-transformed cortisol/DHEA-S ratio (n = 59; β = −0.278, *p* = 0.037), but neither remained significant after FDR correction (q = 0.084 for both); the ratio association was also sensitive to covariate adjustment. In separate mutually adjusted analyses (n = 56), social anxiety remained associated with log IL-6 (partial r = 0.295, *p* = 0.034), whereas the anger-in association was not statistically significant (partial r = −0.026, *p* = 0.858). Cross-equation Wald tests did not detect statistically significant differences between associations with cortisol and log IL-6. **Conclusions:** In this exploratory study, the association between social anxiety and higher IL-6 remained significant after FDR correction across the nine main tests and after separate adjustment for depressive symptom severity. The inverse associations of anger-out with morning cortisol and the cortisol/DHEA-S ratio did not survive FDR correction. These findings warrant longitudinal replication and do not establish causality or distinct biological pathways.

## 1. Introduction

Chronic psychological stress can alter hypothalamic–pituitary–adrenal (HPA) axis activity and inflammatory signaling. The cumulative physiological burden of repeated or poorly regulated stress responses is captured by the concept of allostatic load, which links chronic stress to adverse health outcomes [1]. Understanding the biological correlates of emotional distress requires consideration of both specific emotional experiences and patterns of emotional expression. Examining these characteristics alongside both hormonal and inflammatory biomarkers may help clarify their biological correlates in psychiatric populations.

For anger, distinguishing the experience of emotion from the ways in which it is expressed is particularly relevant. The State-Trait Anger Expression Inventory distinguishes anger directed outward (anger-out), anger held in (anger-in) and the tendency to control anger expression (anger-control) [2]. Associations between anger expression and cortisol have varied across study contexts. In an occupational sample, higher anger-out amplified the association between job strain and early-morning cortisol [3]. Another study found that anger-out was associated with a flatter cortisol awakening response within a subgroup reporting high anger expression [4]. Cultural context may also be relevant: a comparison of American and Japanese samples found opposite associations between anger expression and a composite measure of inflammatory and cardiovascular risk [5]. Where emotional restraint is strongly emphasized, outward anger expression may carry different psychological meanings and interpersonal functions than in contexts where such expression is more socially accepted. In Korea, hwa-byung has been described as a clinical presentation involving chronically suppressed anger [6]. These observations support examining anger expression within its clinical and cultural context.

Social anxiety provides a complementary focus on fear and avoidance in situations involving social evaluation. Epidemiological research has linked anxiety disorders to low-grade inflammation in some patient subgroups, with findings varying by sex and diagnostic subtype [7]. A meta-analysis of generalized anxiety disorder found elevated CRP relative to controls, albeit with a small effect size and marked heterogeneity across studies [8]. Experimental research nevertheless provides a rationale for examining social-evaluative concerns in relation to stress biology. A meta-analysis of laboratory studies found particularly large cortisol responses to stressors combining social-evaluative threat and uncontrollability [9], while an experimental study linked social-evaluative threat to increased stimulated proinflammatory cytokine production [10]. These observations have been formalized in models of psychobiological responses to social threat [11]. In an experimental study, anger and fear responses to a laboratory stressor showed differing associations with cortisol and the inflammatory cytokine interleukin-6 (IL-6) [12]. Whether such laboratory observations extend to habitual anger expression and social anxiety symptoms in psychiatric outpatients remains uncertain.

The choice of hormonal indices also requires consideration. Dehydroepiandrosterone sulfate (DHEA-S) exerts anti-glucocorticoid effects [13], and the cortisol/DHEA-S ratio has therefore been proposed as a measure of integrated HPA-axis output that is less sensitive to single-point variation than cortisol alone; methodological guidance on constructing and interpreting such hormone ratios is available [14]. This ratio has predicted mortality in a large cohort [15] and has been reported to be elevated in drug-free depression [16] and in treatment-resistant depression [17]. Including the ratio alongside cortisol provides a complementary index of HPA-axis-related activity.

Against this background, we conducted an exploratory study to examine the associations of anger expression and social anxiety with hormonal indices (cortisol and the cortisol/DHEA-S ratio) and an inflammatory marker (IL-6) in psychiatric outpatients. Additional analyses assessed selected associations after adjustment for depressive symptom severity and examined the respective associations of anger-in and social anxiety with IL-6 after accounting for one another. We also explored whether the associations of anger-out and social anxiety differed between cortisol and IL-6.

## 2. Materials and Methods

### 2.1. Study Design and Participants

This was a single-center, cross-sectional study based on a retrospective review of clinical records in the Stress Clinic of the Department of Psychiatry of Yonsei University Wonju Severance Christian Hospital. This study is reported in accordance with the STROBE recommendations [18]. Patients attending their initial visit to the clinic between August 2023 and March 2026 were screened for eligibility. The sample size was determined by the number of eligible patients with available data during the study period; no a priori sample size calculation was performed.

**Figure 1:**
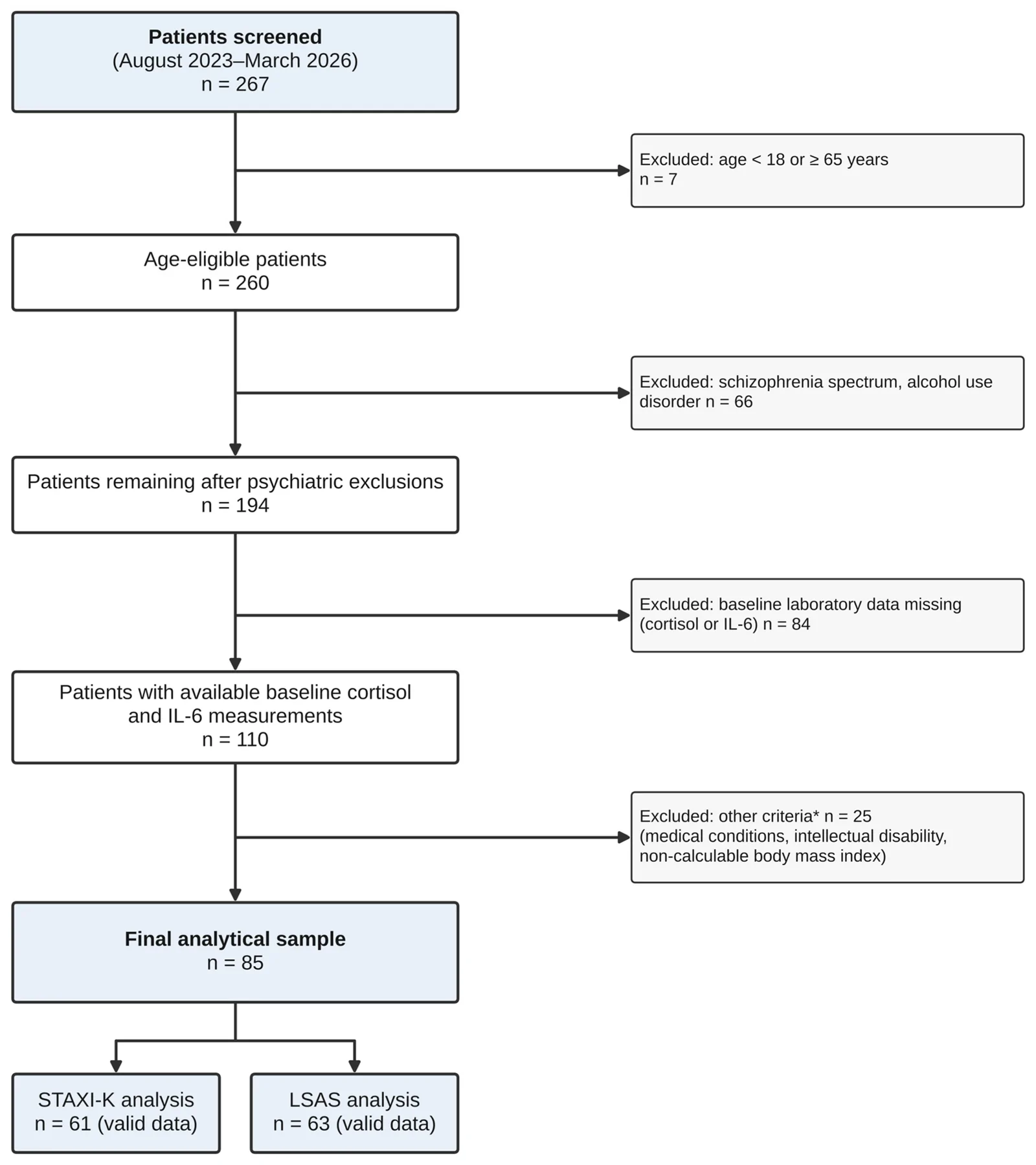
Flow of participants through this study. Exclusion criteria were applied sequentially; the count at each step therefore depends on the order of application, whereas the final sample size does not. * Other criteria comprised malignancy, rheumatic or autoimmune disease, hepatic cirrhosis, structural brain lesion or epilepsy, fibromyalgia, C-reactive protein > 3 mg/dL, intellectual disability and non-calculable body mass index.

The exclusion criteria were applied sequentially. Participants were excluded if they were younger than 18 years or aged 65 years or older; had a schizophrenia-spectrum disorder or alcohol use disorder; lacked baseline cortisol or IL-6 measurements; or had malignancy, rheumatic or autoimmune disease, hepatic cirrhosis, structural brain lesion, epilepsy, fibromyalgia, or C-reactive protein (CRP) concentration > 3 mg/dL. Patients with intellectual disability were excluded because the validity of self-report assessment could not be assured. Patients with a bipolar spectrum disorder (n = 5; one bipolar I disorder without a current mood episode and four bipolar II disorder) were retained, consistent with the dimensional focus of this study. Of 267 patients screened, 85 constituted the final analytical sample (Figure 1). The sample size for each analysis depended on the availability of the required measurements.

Psychiatric diagnoses were obtained from the diagnostic formulation recorded by the attending psychiatrist at the intake visit, when blood sampling was performed. The primary diagnosis was defined as the first diagnosis listed. A comorbid diagnosis was defined as an additional psychiatric condition recorded in the diagnostic formulation that belonged to a diagnostic category other than that of the primary diagnosis; non-psychiatric medical conditions and differential diagnoses qualified as less likely were not counted. This study was conducted in accordance with the Declaration of Helsinki and was approved by the Institutional Review Board of Yonsei University Wonju Severance Christian Hospital (Approval number CR326081; date of approval 16 June 2026). The requirement for informed consent was waived by the Institutional Review Board because this study was a retrospective review of existing clinical records and the blood tests were performed as part of routine clinical assessment at the Stress Clinic.

### 2.2. Measures

**Anger expression.** Anger expression was assessed with the Korean version of the State-Trait Anger Expression Inventory (STAXI-K) [2]. Its three expression subscales, anger-out, anger-in and anger-control, each comprise eight items rated from 1 to 4.

**Social anxiety.** Social anxiety was assessed with the Korean version of the Liebowitz Social Anxiety Scale (LSAS) [19,20], which assesses fear/anxiety and avoidance across 24 situations. The total score was used as the principal measure of social anxiety. Additional analyses examined the fear/anxiety and avoidance dimensions and four situation-type subscales: social interaction, observation, performance and assertion.

**Depressive symptom severity.** Depressive symptom severity was assessed with the Korean version of the nine-item Patient Health Questionnaire (PHQ-9) [21], scored 0–3 per item (total range 0–27).

**Questionnaire administration and missing data.** For the LSAS and STAXI-K, each instrument was introduced into routine intake assessment of the Stress Clinic during the study period; patients whose initial visits preceded its introduction did not receive the corresponding questionnaire. After introduction, occasional administrative omissions in questionnaire distribution resulted in nonadministration for some patients. Item-level missingness within returned questionnaires was handled separately. Where individual items were missing, scale scores were prorated from the mean of the completed items; this applied to the anger-in subscale for two participants (one item each), the LSAS for two participants (two item ratings each) and the PHQ-9 for one participant (one item). Proration was not applied to questionnaires that had not been administered.

### 2.3. Biomarker Assessment

To minimize diurnal variation, all blood samples were drawn before 09:30 after an overnight fast. Serum cortisol, DHEA-S and CRP were measured in the hospital’s Department of Laboratory Medicine. Cortisol was measured by chemiluminescent immunoassay on an Atellica IM 1600 analyzer using the Atellica IM Cortisol reagent (Siemens Healthcare Diagnostics Inc., Tarrytown, NY, USA; analytical measuring range 0.50–75.00 µg/dL). DHEA-S was measured by enzyme immunoassay on a ThunderBolt EIA/CLIA analyzer (Gold Standard Diagnostics, Davis, CA, USA) for samples collected between August 2023 and September 2025 and thereafter by chemiluminescent immunoassay on a LIAISON XL analyzer (DiaSorin, Saluggia, Italy; analytical measuring range 1.0–750 µg/dL). Serum IL-6 was measured at an external reference laboratory by electrochemiluminescence immunoassay on a Cobas e801 analyzer using the Elecsys IL-6 reagent (Roche Diagnostics, Rotkreuz, Switzerland; analytical measuring range 1.5–5000 pg/mL); the limit of detection was 1.50 pg/mL.

CRP was measured by a particle-enhanced immunoturbidimetric assay on a Cobas c702 analyzer using the CRP4 reagent (Roche Diagnostics, Rotkreuz, Switzerland; analytical measuring range 0.06–35 mg/dL). The laboratory’s lower reporting limit for CRP changed from 0.40 to 0.05 mg/dL in January 2025. Because this change complicated comparisons of low-range CRP values across the study period, CRP was retained solely for applying the exclusion criterion of >3 mg/dL and was not included in descriptive summaries or biomarker association analyses.

### 2.4. Statistical Analysis

The main associations were examined using hierarchical multiple linear regression. Anger-out, anger-in and LSAS total scores were evaluated separately in relation to cortisol, the cortisol/DHEA-S ratio and IL-6. Age, sex and body mass index (BMI) were entered at Step 1, and the psychological variable of interest was added at Step 2. BMI was included to account for adiposity-related variation in IL-6 [22]. Unstandardized coefficients, 95% confidence intervals, standardized coefficients and changes in explained variance (ΔR^2^) were reported for the main regression models. Age, sex and BMI were selected as core covariates because of their established associations with cortisol, DHEA-S and IL-6, and the covariate set was kept small relative to the sample size; other potential confounders, including depressive symptom severity, psychotropic exposure, HbA1c, smoking and diagnostic category, were examined in sensitivity analyses.

This study was not preregistered, and no statistical analysis plan specifying the primary hypotheses and testing strategy was documented before the predictor–biomarker associations were examined. The analyses are presented as literature-informed and exploratory, with the main association models distinguished from additional exploratory and sensitivity analyses. The main association models comprised the nine combinations of anger-out, anger-in and LSAS total scores with cortisol, the log-transformed cortisol/DHEA-S ratio and log IL-6. To account for multiplicity across these nine tests, Benjamini–Hochberg false discovery rate (FDR)-adjusted q values are reported alongside nominal *p* values. Sensitivity analyses, LSAS subscale analyses, categorical IL-6 analyses and cross-biomarker comparisons were considered additional exploratory analyses; apart from the Bonferroni correction applied to the LSAS subscales, their *p* values were not adjusted for multiplicity.

Because no a priori sample size calculation was performed, a sensitivity power analysis was conducted. With 59 to 63 participants and three covariates, the hierarchical regression models had 80% power at a two-sided α of 0.05 to detect an increment of f^2^ ≥ 0.14 for the added psychological variable, corresponding to a partial R^2^ of approximately 0.12.

The cortisol/DHEA-S ratio was calculated as cortisol divided by DHEA-S and natural-log transformed. Cortisol was analyzed on its original scale. IL-6 concentrations were below the LOD in 19 participants (22.4%); for the main linear regression analyses, these values were substituted with LOD/√2 before natural-log transformation. DHEA-S, triglycerides and the triglyceride/low-density lipoprotein cholesterol ratio were also natural-log transformed for their respective analyses.

Exploratory Pearson correlations among the principal variables, including anger-control, were calculated using pairwise complete observations (Table S1). Anger-in and social anxiety were strongly correlated (r = 0.77) and were therefore examined in separate main regression models. Their associations with log IL-6 after mutual adjustment were subsequently assessed using partial correlations controlling for age, sex, BMI and the other psychological variable. These analyses were restricted to participants with complete data on both measures and the relevant covariates.

Sensitivity analyses assessed selected associations after the separate addition of PHQ-9 scores or psychotropic exposure to the base covariate set. A further analysis of the social anxiety–IL-6 association excluded participants taking psychotropic medication at blood sampling. Additional exploratory sensitivity analyses re-estimated the four associations that were nominally significant in the main models with further adjustment for HbA1c, which differed between participants with and without STAXI-K data, and for current smoking; after excluding participants with a primary bipolar spectrum diagnosis (n = 5); and with adjustment for broad diagnostic category (depressive, anxiety, trauma- and stressor-related or other disorders). Because DHEA-S was measured on two platforms, the anger-out–ratio association was additionally examined with adjustment for assay platform and after restriction to samples measured before the platform change. Left-censored Tobit models, with the censoring threshold set at log(1.50), were used to assess the social anxiety–IL-6 and anger-in–IL-6 associations while accounting for concentrations below the LOD. These models assumed a normally distributed latent log IL-6 outcome conditional on the covariates. Additional Tobit models included PHQ-9 scores. For LSAS subscale analyses, each subscale was entered into a separate regression model because of the high intercorrelation among subscales (r = 0.69–0.95). A Bonferroni-corrected threshold of α = 0.05/7 ≈ 0.007 was applied across the seven comparisons.

Cross-equation Wald tests within a seemingly unrelated regression framework compared the associations of anger-out and social anxiety with cortisol versus log IL-6. These comparisons directly evaluated differences between coefficients rather than inferring differences from significant and non-significant results in separate models [23].

In a complementary categorical analysis, participants with IL-6 concentrations below 1.50 pg/mL were compared with those with concentrations ≥ 3.00 pg/mL using Mann–Whitney U tests with rank-biserial r as the effect size; participants with intermediate concentrations were excluded from these comparisons. The lower threshold of 1.50 pg/mL was the assay detection limit, whereas 3.00 pg/mL was selected by the authors for an exploratory contrast and was not an established clinical or biological cut-off. Separate logistic regression models on the full sample examined the associations of anger expression phenotypes and LSAS total scores with the odds of a detectable IL-6 concentration (≥1.50 pg/mL). Odds ratios and profile-likelihood 95% confidence intervals were reported.

Regression models used complete cases for the variables required by each model, whereas correlation analyses used pairwise complete observations. Participants were not required to have both LSAS and STAXI-K data for models involving only one of these measures. Analyses requiring both psychological measures were restricted to participants with data on both questionnaires and the relevant covariates. Participants for whom BMI could not be computed because of missing anthropometric data were therefore excluded from the primary analysis; a sensitivity analysis reincluding them, with and without mean substitution of BMI, is reported in the Supplementary Materials. All analyses were performed in R 4.6.1 (R Foundation for Statistical Computing, Vienna, Austria). Left-censored Tobit models were obtained with the survreg function of the survival package (3.8.6), seemingly unrelated regression with systemfit package (1.1.30) and cross-equation Wald tests on standardized coefficients with the linearHypothesis function in the car package (3.1.5). Two-sided *p* values below 0.05 were considered statistically significant.

## 3. Results

### 3.1. Participant Characteristics

Of 267 patients screened, 85 formed the analytical sample after sequential application of the exclusion criteria (Figure 1). The mean age was 36.0 years (SD 12.2), and 48 participants (56.5%) were women. The mean BMI was 24.5 kg/m^2^ (SD 5.0), 24 participants (28.2%) were current smokers, and the mean PHQ-9 total score was 15.0 (SD 8.2). Sixty-one participants (71.8%) reported lifetime psychotropic exposure, of whom 39 (45.9% of the sample) were taking medication at the time of blood sampling. STAXI-K data were available for 61 participants and LSAS data for 63; 56 participants had data on both measures. Two participants with available STAXI-K data had missing DHEA-S measurements, leaving 59 participants for the cortisol/DHEA-S ratio analysis. The most frequent primary diagnoses at intake were depressive disorders (27; 31.8%), anxiety disorders (20; 23.5%) and trauma- and stressor-related disorders (20; 23.5%); 43 participants (50.6%) had at least one comorbid diagnosis. Baseline characteristics are summarized in Table 1.

Comparison of participants with versus without available STAXI-K data showed higher PHQ-9 scores (*p* = 0.030) and higher HbA1c (*p* = 0.037) among participants with available STAXI-K data. Log IL-6 was also numerically higher in this group, although the difference was not statistically significant (*p* = 0.052). No statistically significant differences were observed in age, sex or BMI (Table S2).

### 3.2. Anger Expression, Morning Cortisol and the Cortisol/DHEA-S Ratio

Among participants with available STAXI-K data, higher anger-out scores were associated with lower serum cortisol after adjustment for age, sex and BMI (n = 61; B = −0.403, 95% CI −0.744 to −0.061, β = −0.311, *p* = 0.022, ΔR^2^ = 0.079; Table 2a). Higher anger-out scores were also associated with a lower log-transformed cortisol/DHEA-S ratio (n = 59; B = −0.038, 95% CI −0.074 to −0.002, β = −0.278, *p* = 0.037, ΔR^2^ = 0.062; Table 2b). Both associations remained statistically significant after additional adjustment for depressive symptom severity (cortisol β = −0.355, *p* = 0.011; ratio β = −0.310, *p* = 0.026) and for psychotropic exposure (cortisol β = −0.401, *p* = 0.005; ratio β = −0.359, *p* = 0.011). Neither anger-in nor social anxiety showed a statistically significant association with either hormonal index in the age-, sex-, and BMI-adjusted models (all *p* ≥ 0.244). Neither anger-out association remained significant after FDR correction across the nine main association tests (q = 0.084; Section 3.3).

### 3.3. Anger Suppression, Social Anxiety and Inflammation

With log IL-6 as the outcome, anger-in and social anxiety were examined in separate models adjusted for age, sex, and BMI. Higher anger-in scores were associated with higher log IL-6 (n = 61; B = 0.037, 95% CI 0.003 to 0.071, β = 0.294, *p* = 0.033, ΔR^2^ = 0.068; Table 3a). Higher LSAS total scores were also associated with higher log IL-6 (n = 63; B = 0.007, 95% CI 0.003 to 0.011, β = 0.400, *p* = 0.001, ΔR^2^ = 0.141; Table 3b), with the final model explaining 27.0% of the variance in log IL-6.

After Benjamini–Hochberg FDR correction across the nine main association tests (Table 4), the social anxiety–IL-6 association remained significant (q = 0.013), whereas the associations of anger-out with cortisol and with the cortisol/DHEA-S ratio and of anger-in with log IL-6 did not (q = 0.084 for each).

The social anxiety–IL-6 association persisted after separate additional adjustment for depressive symptom severity (*p* = 0.002) or psychotropic exposure (*p* < 0.001), in an analysis restricted to participants not taking psychotropic medication at blood sampling (n = 34, *p* = 0.001), in a left-censored Tobit model (*p* < 0.001), and in a Tobit model additionally adjusted for depressive symptom severity (*p* = 0.002). However, the anger-in–IL-6 association did not reach statistical significance after additional adjustment for depressive symptom severity (*p* = 0.073; Table 5).

Anger-in and LSAS total scores were strongly correlated (r = 0.77; Table S1). Among participants with complete data on both measures and the relevant covariates (n = 56), social anxiety remained associated with log IL-6 after controlling for age, sex, BMI and anger-in (partial r = 0.295, *p* = 0.034). In the corresponding analysis controlling for age, sex, BMI and social anxiety, anger-in was not significantly associated with log IL-6 (partial r = −0.026, *p* = 0.858; Table 5).

Among LSAS subscales, the assertion subscale had the largest standardized coefficient in the separate regression models (β = 0.439, ΔR^2^ = 0.173, *p* < 0.001), followed by social interaction (β = 0.409, *p* = 0.001), fear/anxiety (β = 0.398, *p* = 0.002), avoidance (β = 0.373, *p* = 0.003) and observation (β = 0.358, *p* = 0.004). These five associations met the specified Bonferroni-corrected significance threshold of α ≈ 0.007. The performance subscale did not meet the corrected threshold (β = 0.305, *p* = 0.016). Full subscale results are given in Table S3.

### 3.4. Associations Across Biomarkers

Table 4 summarizes the associations of anger-out, anger-in and social anxiety with the three biomarker outcomes. Anger-out was not significantly associated with log IL-6 (β = −0.031, *p* = 0.824), and neither anger-in nor social anxiety was significantly associated with cortisol or the log-transformed cortisol/DHEA-S ratio. Additional exploratory analyses did not identify significant associations of social anxiety with DHEA-S (*p* = 0.988) or the log triglyceride/low-density lipoprotein cholesterol ratio (*p* = 0.963). The non-significant associations had increments in explained variance of 0.020 or less.

Cross-equation Wald tests comparing the coefficients for cortisol and log IL-6 did not identify statistically significant differences for anger-out (χ^2^(1) = 2.50, *p* = 0.114) or social anxiety (χ^2^(1) = 2.47, *p* = 0.116; Table 5).

**Table 4:** Standardized coefficients (*p*-values) for each predictor–biomarker combination.

Predictor	Cortisol	log Cortisol/DHEA-S	log IL-6
STAXI-K anger-out	−0.311 (0.022) *	−0.278 (0.037) *	−0.031 (0.824)
STAXI-K anger-in	−0.089 (0.528)	−0.159 (0.244)	0.294 (0.033) *
LSAS total	0.140 (0.292)	0.130 (0.306)	0.400 (0.001) *

Each cell reports the standardized coefficient with the *p*-value in parentheses from a separate hierarchical regression adjusted for age, sex and BMI. * *p* < 0.05. Cross-equation Wald tests were not significant. Benjamini–Hochberg false discovery rate-adjusted q values across the nine models were 0.013 for social anxiety with log IL-6; 0.084 for anger-out with cortisol and with the cortisol/DHEA-S ratio and for anger-in with log IL-6; and ≥0.39 for the remaining five associations.

### 3.5. Sensitivity Analyses

Detailed primary and comorbid diagnoses at intake, which informed the diagnostic-category sensitivity analysis, are given in Supplementary Table S4. Additional analyses are summarized in Table 5 and Supplementary Tables S5–S7. The results of adjustment for depressive symptom severity and psychotropic exposure, as well as analyses accounting for the overlap between anger-in and social anxiety, have been described in Section 3.2 and Section 3.3. The remaining sensitivity analyses are presented below.

Reinclusion of participants excluded because BMI could not be calculated did not change the direction or nominal statistical significance of the four associations examined: anger-out with cortisol and the log-transformed cortisol/DHEA-S ratio, and anger-in and social anxiety with log IL-6. These findings were observed both when missing BMI values were replaced with the sample mean and when BMI was omitted from the covariate set (Table S5).

The four nominally significant associations were further examined in additional exploratory sensitivity analyses (Table S6). The associations of anger-out with cortisol, anger-in with log IL-6 and social anxiety with log IL-6 remained significant after additional adjustment for HbA1c or current smoking, after exclusion of the five participants with a primary bipolar spectrum diagnosis and after adjustment for broad diagnostic category; the anger-in–IL-6 association also remained significant after adjustment for lifetime psychotropic exposure (*p* = 0.023). The anger-out–ratio association was in the same direction in all models but was not significant after adjustment for HbA1c (*p* = 0.064), current smoking (*p* = 0.065) or diagnostic category (*p* = 0.068) and was borderline after exclusion of bipolar spectrum diagnoses (*p* = 0.050). Only two samples in this analysis were measured after the DHEA-S platform change; adjustment for assay platform did not change the association (β = −0.278, *p* = 0.039), and the estimate was similar when these two samples were excluded (n = 57, β = −0.265, *p* = 0.051). In left-censored Tobit models, the anger-in–IL-6 association (β = 0.315, *p* = 0.017) was similar to that in the main model and, as in the main analysis, was not significant after additional adjustment for depressive symptom severity (*p* = 0.067; Table S7).

**Table 5:** Sensitivity analyses for the principal associations.

Model/Sensitivity Condition	n	B (95% CI)	Estimate	*p*
Anger-out → cortisol				
Base model (age, sex, BMI)	61	−0.403 (−0.744, −0.061)	β = −0.311	0.022
+PHQ-9	61	−0.459 (−0.809, −0.110)	β = −0.355	0.011
+lifetime psychotropic exposure	61	−0.519 (−0.872, −0.165)	β = −0.401	0.005
Anger-out → log cortisol/DHEA-S	n	B (95% CI)	Estimate	*p*
Base model	59	−0.038 (−0.074, −0.002)	β = −0.278	0.037
+PHQ-9	59	−0.043 (−0.080, −0.005)	β = −0.310	0.026
+lifetime psychotropic exposure	59	−0.049 (−0.087, −0.012)	β = −0.359	0.011
Social anxiety → log IL-6	n	B or partial r (95% CI)	Estimate	*p*
Base model	63	0.007 (0.003, 0.011)	β = 0.400	0.001
+PHQ-9	63	0.008 (0.003, 0.013)	β = 0.474	0.002
+lifetime psychotropic exposure	63	0.007 (0.003, 0.011)	β = 0.421	<0.001
Participants on medication at sampling excluded	34	0.009 (0.004, 0.014)	β = 0.533	0.001
Partial correlation, adjusting for anger-in	56	r = 0.295 (0.024, 0.526)	(-)	0.034
Tobit model, left-censored at the LOD	63	0.007 (0.003, 0.011)	β = 0.413	<0.001
Tobit model + PHQ-9	63	0.008 (0.003, 0.013)	β = 0.461	0.002
Anger-in → log IL-6	n	B or partial r (95% CI)	Estimate	*p*
Base model	61	0.037 (0.003, 0.071)	β = 0.294	0.033
+PHQ-9	61	0.037 (−0.004, 0.078)	β = 0.296	0.073
Partial correlation, adjusting for social anxiety	56	r = −0.026 (−0.296, 0.249)	(-)	0.858
Cross-equation Wald tests (seemingly unrelated regression)	n	Δβ (95% CI)	χ^2^ (1)	*p*
Anger-out: cortisol vs. log IL-6	61	−0.281 (−0.629, 0.067)	2.50	0.114
Social anxiety: log IL-6 vs. cortisol	63	0.259 (−0.064, 0.583)	2.47	0.116

β, standardized coefficient; r, partial correlation coefficient. Dashes indicate that a standardized coefficient is not applicable to partial correlations, for which r is itself a standardized measure of association. LOD, limit of detection.

### 3.6. Categorical Analyses of IL-6

In an exploratory categorical analysis, participants with IL-6 concentrations below 1.50 pg/mL were compared with those with concentrations ≥ 3.00 pg/mL; participants with intermediate concentrations were excluded. Participants in the ≥3.00 pg/mL group had higher anger-in scores (median 22.00 vs. 13.29; U = 31.0, *p* = 0.001, r = 0.730) and higher LSAS total scores (median 93.00 vs. 27.50; U = 33.0, *p* < 0.001, r = 0.761), whereas the between-group difference in anger-out scores was not statistically significant (*p* = 0.125; Table 6).

Separate logistic regression models included participants across the full IL-6 range with complete data and were adjusted for age, sex and BMI. Each one-point increase in anger-in was associated with higher odds of detectable IL-6 (≥1.50 pg/mL; n = 61; OR = 1.250, 95% CI 1.052–1.554, *p* = 0.021). In the separate LSAS model, each one-point increase in LSAS total score was associated with higher odds of detectable IL-6 (n = 63; OR = 1.050, 95% CI 1.019–1.102, *p* = 0.012; Table 7).

## 4. Discussion

In this exploratory cross-sectional study of psychiatric outpatients, anger-out was associated with lower morning serum cortisol and a lower cortisol/DHEA-S ratio, while social anxiety was associated with higher serum IL-6. These associations persisted after separate adjustment for depressive symptom severity or psychotropic exposure. In analyses accounting for the overlap between anger-in and social anxiety, social anxiety remained associated with IL-6, whereas the corresponding anger-in association was not statistically significant. The social anxiety–IL-6 association also remained significant when the analysis was restricted to participants not taking psychotropic medication at sampling and when left-censored Tobit models were used. After FDR correction across the nine main association tests, only the social anxiety–IL-6 association remained significant, so the anger-related associations should be regarded as preliminary. The anger-out–ratio association was also sensitive to covariate specification and was not significant in several sensitivity analyses. Because the data are cross-sectional, none of these associations indicate that anger expression or social anxiety alters biomarker concentrations.

The inverse association between anger-out and cortisol, which did not remain significant after FDR correction, should be interpreted cautiously and in light of differences in study populations, stress contexts and cortisol measures. In an occupational sample, higher anger-out amplified the association between job strain and early-morning cortisol [3], whereas another study found an association between anger-out and a flatter cortisol awakening response within a subgroup reporting high anger expression [4]. These findings are not directly equivalent to an association with a single morning serum cortisol measurement. One possible explanatory framework is a model of stress-related hypocortisolism, which proposes that prolonged HPA-axis activation may be followed by reduced cortisol activity [24]. However, the present association reflects relative differences within a clinical sample and does not establish hypocortisolism, impaired stress reactivity or a preceding period of HPA-axis hyperactivation. The association may also reflect shared influences, such as current stress exposure, medication or overall symptom burden, and does not indicate that anger expression affects HPA-axis activity. The cortisol/DHEA-S ratio provides a complementary hormonal measure, but its interpretation requires consideration [14]. Because cortisol is its numerator, the ratio association does not provide independent confirmation of HPA-axis hypofunction.

Cultural context provides another consideration. Where emotional restraint is strongly emphasized, outward anger expression may carry different psychological meanings and interpersonal functions than in settings where such expression is more socially accepted. The cross-cultural finding that anger expression carries opposite health correlates in different cultural contexts supports treating expression style as context-dependent rather than universally interpretable [5]. The present sample was recruited in Korea, but cultural moderation was not tested. Replication across cultural settings is therefore needed before attributing the observed association to cultural differences.

The association between social anxiety and IL-6 may be considered within the social signal transduction theory of depression, which proposes that experiences of social threat and adversity can upregulate inflammatory signaling [25]. Social anxiety involves the anticipation of negative social evaluation, providing a plausible conceptual link to this framework without directly testing the proposed mechanism. Experimentally, negative social evaluation rather than mere social presence elicits a cortisol response [26], supporting the physiological relevance of evaluative threat, although acute stress responses and single morning biomarker concentrations represent different aspects of stress biology. The LSAS subscale analyses showed positive associations with IL-6 across several dimensions of social anxiety. The assertion subscale had the largest standardized coefficient, and such situations, which require expressing disagreement or standing one’s ground, arguably represent the most direct form of interpersonal evaluative threat. That fear and avoidance dimensions showed comparable effect sizes suggests that the internal experience of anxiety and its behavioral expression are similarly implicated, rather than one being reducible to the other. However, the highly correlated subscales were analyzed separately, and differences between their coefficients were not formally tested. Therefore, these findings do not establish that assertion-related situations are biologically predominant or that fear/anxiety and avoidance make independent contributions.

The finding that anger suppression lost its association with IL-6 once social anxiety was controlled has a methodological as well as a substantive implication. Anger-in and social anxiety were strongly correlated in this sample, and studies examining either construct alone risk attributing to it an association that belongs to the other. In this sample, when the two were examined together, social anxiety showed the stronger association with IL-6. Meanwhile, adjustment for depressive symptom severity showed that the anger-in coefficient was almost unchanged, but its confidence interval widened to include zero. This change in statistical significance should not be interpreted as attenuation of the estimated association by depressive symptoms. However, the present findings should also be considered alongside a cohort study reporting lower IL-6 among participants with social phobia than among those with other anxiety disorders [7]. Diagnostic group comparisons and dimensional associations with symptom severity address different questions. The current results therefore do not imply that patients with social anxiety disorder have higher IL-6 than other diagnostic groups, and further research is needed to clarify how diagnostic status and symptom severity relate to inflammatory markers.

The most important caution concerns what the overall pattern does and does not establish. Cross-equation comparisons did not provide sufficient evidence that the associations differed between cortisol and log IL-6. This does not establish equivalence across biomarkers, but neither does the contrast between significant and non-significant findings in separate models demonstrate biomarker specificity [23]. The observed pattern therefore provides a basis for further investigation rather than evidence of distinct biological pathways. A further caveat concerns IL-6 itself, which is increasingly recognized as a signal of several immunometabolic processes rather than of inflammation alone [27].

Several limitations qualify these findings. First, the cross-sectional design precludes causal inference; reverse causation and residual confounding cannot be excluded. Second, cortisol was sampled at a single morning time point, which cannot capture the cortisol awakening response, diurnal slope or reactivity, although restricting sampling to before 09:30 limited diurnal variability. Third, medication-related sensitivity analyses were limited to lifetime psychotropic exposure and current psychotropic use at blood sampling. Drug class and dose were not consistently documented in the clinical records, limiting the assessment of medication-specific effects on cortisol and IL-6. Although patients with rheumatic or autoimmune disease or malignancy were excluded, these criteria did not directly exclude the use of anti-inflammatory or immunomodulatory medications. Residual medication-related confounding therefore remains possible. Fourth, questionnaire data were not available for all participants because the LSAS and STAXI-K were introduced into routine intake assessment during the study period, and some administrations were missed because of administrative omissions. Participants with available STAXI-K data had higher depressive symptom severity and HbA1c than those without STAXI-K data. These differences raise concerns about the representativeness of the questionnaire subsample. Although separate adjustment for PHQ-9 and HbA1c examined sensitivity to these measured characteristics, it did not resolve the potential effects of selective questionnaire availability. The findings may therefore not fully represent all eligible outpatients, and the direction and magnitude of any resulting selection bias remain uncertain. Fifth, the sample was heterogeneous with respect to diagnosis and included patients with bipolar spectrum disorders whose mood state was not controlled, limiting diagnosis- and mood-state-specific interpretation. Sixth, the sample was modest. In the sensitivity power analysis, the regression models were powered to detect increments in explained variance corresponding to a partial R^2^ of approximately 0.12. The anger-out and anger-in associations (partial R^2^ = 0.08 to 0.09) fell below this threshold, and their 95% confidence intervals extended close to zero (for example, B = −0.403, 95% CI −0.744 to −0.061 for anger-out and cortisol); these estimates are therefore imprecise and may overstate the true associations. The social anxiety–IL-6 association (partial R^2^ = 0.16) exceeded this threshold. Seventh, the sample was drawn from a single center in Korea and was diagnostically heterogeneous, with approximately half of the participants having at least one comorbid diagnosis. Therefore, the findings describe dimensional associations in a mixed outpatient sample and should not be generalized to specific psychiatric diagnoses or to other cultural and clinical populations without replication.

Measurement-related limitations also warrant consideration. DHEA-S was measured on two different platforms across the study period, and potential between-platform differences may have influenced both the DHEA-S concentrations and the cortisol/DHEA-S ratio. Only two samples in the ratio analysis were measured after the platform change, so between-platform differences could not be meaningfully tested; adjusting for platform or excluding these two samples did not materially change the anger-out–ratio association. In addition, 22.4% of IL-6 measurements were below the detection limit. Although the social anxiety association remained significant in left-censored Tobit models, these analyses relied on distributional assumptions and did not eliminate all uncertainty associated with low-concentration measurements. IL-6 was also the only inflammatory marker analyzed: CRP could not be used because its reporting limit changed during the study period, and no other cytokines were measured, so the findings may not extend to other inflammatory indices. Finally, multiple predictor–biomarker and secondary analyses were performed, increasing the possibility of chance findings. Only the social anxiety–IL-6 association remained significant after FDR correction across the nine main association tests, and the additional analyses were not adjusted for multiplicity; the findings should therefore be regarded as hypothesis-generating. Larger longitudinal studies with repeated measurements and clearly specified primary analyses are needed to assess reproducibility, temporal direction and clinical relevance.

## 5. Conclusions

In this exploratory cross-sectional study of psychiatric outpatients, higher social anxiety was associated with higher serum IL-6, which was the only association that remained significant after FDR correction across the nine main tests. This association also persisted in the reported sensitivity analyses and after adjustment for anger-in in a separate analysis. Higher anger-out was associated with lower morning cortisol and a lower cortisol/DHEA-S ratio, but neither association remained significant after FDR correction. These findings suggest that specific emotional characteristics warrant further investigation in relation to stress-related biomarkers, although they do not establish causality or distinct neuroendocrine and inflammatory pathways. Larger longitudinal studies with repeated biomarker assessments are needed to evaluate the reproducibility and temporal relationships of these associations.

## Figures and Tables

**Table 1:** Baseline characteristics of the study participants.

Characteristics	Mean (SD) or n (%)
Demographic characteristics	
Age, years	36.0 (12.2)
Female/male, n (%)	48 (56.5)/37 (43.5)
Body mass index, kg/m^2^	24.5 (5.0)
Current smoker, n (%)	24 (28.2)
Clinical characteristics	
PHQ-9 total score	15.0 (8.2)
Any lifetime psychotropic exposure, n (%)	61 (71.8)
Taking medication at blood sampling, n (%)	39 (45.9)
LSAS total score (n = 63)	64.2 (41.6)
STAXI-K anger-in (n = 61)	20.0 (5.7)
STAXI-K anger-out (n = 61)	15.8 (5.4)
STAXI-K anger-control (n = 61)	20.2 (5.1)
Biomarkers	
Cortisol, µg/dL	16.9 (7.3)
DHEA-S, µg/dL (n = 82)	213.8 (133.3)
IL-6, pg/mL ^a^	3.1 (3.0)
IL-6 ≥ LOD, n (%)	66 (77.6)
HbA1c, %	5.6 (0.7)
Primary diagnosis at intake, n (%)	
Depressive disorders	27 (31.8)
Anxiety disorders	20 (23.5)
Trauma- and stressor-related disorders	20 (23.5)
Bipolar spectrum disorders	5 (5.9)
Somatic symptom-related disorders	5 (5.9)
Other ^b^	8 (9.4)
At least one comorbid diagnosis	43 (50.6)

Values are mean (SD) unless otherwise indicated. Summary statistics are based on available observations. STAXI-K data were unavailable for 24 participants, LSAS data for 22, and DHEA-S data for 3; all other listed variables were complete. ^a^ The IL-6 mean incorporates LOD/√2 substituted values. ^b^ Other comprises attention-deficit/hyperactivity disorder (n = 3), obsessive-compulsive-related disorders (n = 2), and pathological gambling, an eating disorder and depersonalization/derealization disorder (n = 1 each). DHEA-S, dehydroepiandrosterone sulfate; IL-6, interleukin-6; LOD, limit of detection; LSAS, Liebowitz Social Anxiety Scale; PHQ-9, Patient Health Questionnaire-9; STAXI-K, State-Trait Anger Expression Inventory, Korean version.

**Table 2:** Hierarchical regression of morning cortisol and the cortisol/DHEA-S ratio on anger-out. (**a**) Serum cortisol. (**b**) Log cortisol/DHEA-S ratio.

(**a**) Serum cortisol (n = 61).					
Predictor	*B*	SE	95% CI	β	*p*
Step 1: Covariates (*R*^2^ = 0.126; *F*(3,57) = 2.75, *p* = 0.051)					
Age	0.042	0.073	−0.104, 0.188	0.075	0.567
Sex (male)	3.165	1.798	−0.435, 6.765	0.228	0.084
Body mass index	−0.351	0.157	−0.665, −0.037	−0.278	0.029
Step 2: Anger-out added (*R*^2^ = 0.206; Δ*R*^2^ = 0.079, Δ*F p* = 0.022)					
Age	0.024	0.071	−0.118, 0.165	0.042	0.738
Sex (male)	4.610	1.835	0.935, 8.285	0.331	0.015
Body mass index	−0.276	0.154	−0.585, 0.033	−0.218	0.079
STAXI-K anger-out	−0.403	0.170	−0.744, −0.061	−0.311	0.022
(**b**) Log cortisol/DHEA-S ratio (n = 59).					
Predictor	*B*	SE	95% CI	β	*p*
Step 1: Covariates (*R*^2^ = 0.198; *F*(3,55) = 4.54, *p* = 0.006)					
Age	0.014	0.008	−0.002, 0.029	0.218	0.086
Sex (male)	−0.345	0.186	−0.719, 0.028	−0.231	0.069
Body mass index	−0.033	0.016	−0.065, 0.000	−0.245	0.048
Step 2: Anger-out added (*R*^2^ = 0.261; Δ*R*^2^ = 0.062, Δ*F p* = 0.037)					
Age	0.012	0.008	−0.004, 0.027	0.185	0.137
Sex (male)	−0.202	0.193	−0.588, 0.185	−0.135	0.300
Body mass index	−0.026	0.016	−0.059, 0.006	−0.197	0.105
STAXI-K anger-out	−0.038	0.018	−0.074, −0.002	−0.278	0.037

B, unstandardized coefficient; CI, confidence interval; β, standardized coefficient. Two participants with missing DHEA-S values were excluded from the ratio analysis (**b**).

**Table 3:** Hierarchical regression of log IL-6. (**a**) STAXI-K anger-in. (**b**) LSAS total score.

(**a**) STAXI-K anger-in (n = 61).					
Predictor	B	SE	95% CI	β	*p*
Step 1: Covariates (*R*^2^ = 0.142; *F*(3,57) = 3.14, *p* = 0.032)					
Age	−0.012	0.007	−0.027, 0.003	−0.209	0.108
Sex (male)	0.165	0.184	−0.204, 0.535	0.115	0.373
Body mass index	0.033	0.016	0.001, 0.065	0.253	0.045
Step 2: Anger-in added (*R*^2^ = 0.210; Δ*R*^2^ = 0.068, Δ*F p* = 0.033)					
Age	−0.005	0.008	−0.021, 0.011	−0.091	0.505
Sex (male)	0.155	0.179	−0.203, 0.512	0.107	0.390
Body mass index	0.025	0.016	−0.007, 0.057	0.192	0.121
STAXI-K anger-in	0.037	0.017	0.003, 0.071	0.294	0.033
(**b**) LSAS total score (n = 63).					
Predictor	B	SE	95% CI	β	*p*
Step 1: Covariates (*R*^2^ = 0.129; *F*(3,59) = 2.91, *p* = 0.042)					
Age	−0.006	0.008	−0.022, 0.009	−0.108	0.403
Sex (male)	0.210	0.189	−0.168, 0.588	0.142	0.270
Body mass index	0.040	0.016	0.008, 0.072	0.301	0.016
Step 2: LSAS total added (*R*^2^ = 0.270; Δ*R*^2^ = 0.141, Δ*F p* = 0.001)					
Age	−0.003	0.007	−0.017, 0.011	−0.048	0.688
Sex (male)	0.189	0.174	−0.160, 0.538	0.128	0.284
Body mass index	0.024	0.016	−0.007, 0.056	0.182	0.127
LSAS total	0.007	0.002	0.003, 0.011	0.400	0.001

Outcome: natural-log transformed serum IL-6 (pg/mL). Values below the limit of detection (LOD) were substituted with LOD/√2 before transformation. LSAS, Liebowitz Social Anxiety Scale.

**Table 6:** Comparison of extreme IL-6 groups (Mann–Whitney U tests).

Predictor	n (Low/High)	Median (IQR), Low vs. High	*U*	*p*	*r*
STAXI-K anger-in	10/23	13.29 (12.00–17.75) vs. 22.00 (19.00–25.07)	31.0	0.001	0.730
STAXI-K anger-out	10/23	12.50 (10.00–16.25) vs. 16.00 (13.50–20.00)	75.5	0.125	0.343
LSAS total	12/23	27.50 (16.18–29.75) vs. 93.00 (53.00–121.00)	33.0	<0.001	0.761

Low, IL-6 < 1.50 pg/mL; high, IL-6 ≥ 3.00 pg/mL. Participants whose values fell in the intermediate range were excluded, so group sizes differ between predictors according to questionnaire completion. r denotes the rank-biserial effect size.

**Table 7:** Logistic regression for detectable IL-6 (≥limit of detection).

Predictor	OR	95% CI	*p*
STAXI-K anger-in (n = 61)	1.250	1.052, 1.554	0.021
LSAS total (n = 63)	1.050	1.019, 1.102	0.012

Adjusted for age, sex and BMI. OR, odds ratio; CI, profile-likelihood confidence interval.

## Data Availability

The data presented in this study are available on request from the corresponding author due to privacy and ethical reasons, as they contain clinical information from psychiatric patients.

## Supplementary Materials

The following supporting information can be downloaded at: https://www.mdpi.com/article/10.3390/brainsci16101134/s1, STROBE checklist for cross-sectional studies; Table S1: Pearson correlation matrix of the principal variables; Table S2: Baseline characteristics of participants with versus without available STAXI-K data; Table S3: LSAS subscales predicting log IL-6; Table S4: Detailed distribution of diagnoses at intake; Table S5: Sensitivity analysis reincluding participants with non-calculable BMI; Table S6: Additional sensitivity analyses of the four associations that were nominally significant in the main models; Table S7: Left-censored Tobit models for log IL-6.
