# Supplementary material for: Associations of Anger Expression and Social Anxiety with Cortisol, the Cortisol/DHEA-S Ratio, and Interleukin-6 in Psychiatric Outpatients: An Exploratory Cross-Sectional Study
Source: Brain Sci. 2026 Oct 20;16(10):1134. doi: 10.3390/brainsci16101134 (PMC13702419; doi:10.3390/brainsci16101134)
Supplement: Supplementary file 1 [file brainsci-16-01134-s001.zip › brainsci-4611488-supplementary.pdf]

Supplementary materials

STROBE Statement - Checklist for cross-sectional studies

This study was reported in accordance with the STROBE (Strengthening the Reporting of Observational Studies in Epidemiology) statement. The table below lists the 22 checklist items and indicates where each is addressed.

No.	**Item**	**Recommendation**	**Location**
Title and abstract			
1	Title and abstract	(a) Indicate the study design with a commonly used term in the title or abstract. (b) Provide in the abstract with an informative and balanced summary of what was done and what was found.	Title; Abstract
Introduction			
2	Background/rationale	Explain the scientific background and rationale for the investigation being reported.	Section 1, paragraphs 1–4
3	Objectives	State specific objectives, including any prespecified hypotheses.	Section 1, final paragraph
Methods			
4	Study design	Present key elements of study design early in the paper.	Section 2.1
5	Setting	Describe the setting, locations, and relevant dates, including periods of recruitment and data collection.	Section 2.1 (Aug 2023–Mar 2026)
6	Participants	Give the eligibility criteria, and the sources and methods of selection of participants.	Section 2.1; Figure 1
7	Variables	Clearly define all outcomes, exposures, predictors, potential confounders and effect modifiers.	Sections 2.2–2.4
8	Data sources/measurement	For each variable of interest, give sources of data and details of methods of assessment.	Sections 2.2, 2.3
9	Bias	Describe any efforts to address potential sources of bias.	Section 2.4; Section 4 (limitations)
10	Study size	Explain how the study size was arrived at.	Section 2.1 (all available cases)
11	Quantitative variables	Explain how quantitative variables were handled in the analyses.	Section 2.4 (LOD/√2, log transformation, Tobit)
12	Statistical methods	(a) All statistical methods. (b) Subgroup and interaction analyses. (c) Handling of missing data. (e) Sensitivity analyses.	Section 2.4; Table S5
Results			
13	Participants	Numbers at each stage, reasons for non-participation.	Figure 1
14	Descriptive data	Characteristics of study participants and number with missing data for each variable.	Section 3.1; Table 1
15	Outcome data	Report numbers of outcome events or summary measures.	Table 1; Sections 3.2–3.6
16	Main results	(a) Unadjusted and confounder-adjusted estimates with precision (95% CI). (b) Category boundaries. (c) Absolute risk where relevant.	Tables 2–7
17	Other analyses	Subgroup, interaction and sensitivity analyses.	Sections 3.3–3.6; Tables S2, S5

Discussion			
18	Key results	Summarize key results with reference to study objectives.	Section 4, paragraph 1
19	Limitations	Discuss limitations, considering sources of potential bias or imprecision.	Section 4, last two paragraphs
20	Interpretation	Give a cautious overall interpretation considering objectives, limitations, multiplicity of analyses and other relevant evidence.	Section 4
21	Generalizability	Discuss the external validity of the study results.	Section 4 (limitations)
Other information			
22	Funding	Give the source of funding and the role of the funders.	Funding statement

Item locations refer to sections of the main manuscript. Items with multiple sub-recommendations (8, 12, 16, 17) are addressed in the sections indicated.

Table S1. Pearson correlation matrix of the principal variables.

Variable	1	2	3	4	5	6	7	8	9
1. STAXI-K anger-in	—								
2. STAXI-K anger-out	0.54***	—							
3. STAXI-K anger-control	-0.25	-0.57***	—						
4. LSAS total	0.77***	0.42**	-0.34*	—					
5. Body mass index	0.23	0.20	-0.07	0.27*	—				
6. Age	-0.42***	-0.21	-0.07	-0.26	-0.07	—			
7. Cortisol	-0.13	-0.24	0.07	0.01	-0.28*	0.03	—		
8. DHEA-S	0.25	0.24	0.11	0.08	0.06	-0.35**	0.25	—	
9. Log IL-6	0.39**	0.11	-0.01	0.46***	0.27*	-0.26*	0.06	0.14	—

Correlations were computed among participants with STAXI-K data. Only the lower triangle is shown; significance levels are uncorrected two-tailed p values. Values are Pearson r. * $p < 0.05$, ** $p < 0.01$, *** $p < 0.001$. Pairwise complete observations were used. The two pairs at greatest risk of collinearity (anger-in with LSAS, r = 0.77; anger-out with anger-control, r = -0.57) were modeled in separate regressions.

Table S2. Baseline characteristics of participants with versus without available STAXI-K data.

Variable	With STAXI-K (n = 61)	Without STAXI-K (n = 24)	Test	*p*
Demographic characteristics				
Age, years	36.6 (12.3)	34.7 (12.1)	U = 809	0.455
Body mass index, kg/m^2	25.0 (5.5)	23.0 (3.2)	U = 855.5	0.230
Female, n (%)	35 (57.4)	13 (54.2)	$\chi^2(1)$ =	0.979

Variable	With STAXI-K (n = 61)	Without STAXI-K (n = 24)	Test	*p*
			0.001	
Current smoker, n (%)	18 (29.5)	6 (25.0)	$\chi^2(1)$ = 0.022	0.882
Clinical characteristics				
PHQ-9 total score	16.1 (8.4)	12.0 (7.1)	U = 954.5	0.030
Biomarkers				
Cortisol, µg/dL	17.5 (6.9)	15.4 (8.2)	U = 898.5	0.105
Log IL-6	0.94 (0.72)	0.63 (0.57)	U = 930.5	0.052
HbA1c, %	5.7 (0.7)	5.4 (0.4)	U = 945.5	0.037
IL-6 ≥ LOD, n (%)	51 (83.6)	15 (62.5)	$\chi^2(1)$ = 3.29	0.070

Values are mean (SD) or n (%). Continuous variables were compared with the Mann–Whitney U test because of departures from normality, and categorical variables were compared with the chi-square test.

Table S3. LSAS subscales predicting log IL-6 (n = 63).

LSAS subscale	B	SE	β	p	ΔR^2	95% CI	Bonf.
Response dimensions							
Fear/anxiety	0.013	0.004	0.398	0.002	0.138	0.005, 0.021	Pass
Avoidance	0.013	0.004	0.373	0.003	0.126	0.005, 0.021	Pass
Situation types							
Social interaction	0.019	0.006	0.409	0.001	0.145	0.008, 0.030	Pass
Observation	0.021	0.007	0.358	0.004	0.117	0.007, 0.034	Pass
Performance	0.027	0.011	0.305	0.016	0.084	0.005, 0.048	Fail
Assertion	0.052	0.014	0.439	<0.001	0.173	0.025, 0.080	Pass

The response dimensions (fear/anxiety and avoidance) and the four situation types were each entered into a separate hierarchical regression adjusted for age, sex and body mass index. Because the subscales were highly intercorrelated (r = 0.69–0.95), they were not entered simultaneously, and a Bonferroni-corrected threshold of α = 0.007 was applied across the seven comparisons. β and ΔR^2 are taken from Step 2. Bonf., outcome of Bonferroni correction at α = 0.007.

Table S4. Detailed distribution of diagnoses at intake (n = 85).

Diagnoses were classified from the diagnostic formulation recorded at the intake visit, at which blood sampling was performed. The primary diagnosis was defined as the first diagnosis listed. A comorbid diagnosis was defined as an additional psychiatric condition recorded in the diagnostic formulation that belonged to a diagnostic category other than that of the primary diagnosis. Consistent with intake practice, provisional (rule-out) formulations were treated as diagnoses.

(a) Primary diagnosis

Primary diagnosis at intake	n	%
**Depressive disorders**	**27**	**31.8**
Major depressive disorder	12	14.1

**Primary diagnosis at intake**	**n**	**%**
Persistent depressive disorder	8	9.4
Depressive disorder, unspecified	7	8.2
**Anxiety disorders**	**20**	**23.5**
Panic disorder	9	10.6
Anxiety disorder, unspecified	7	8.2
Social anxiety disorder	2	2.4
Generalized anxiety disorder	2	2.4
**Trauma- and stressor-related disorders**	**20**	**23.5**
Adjustment disorder	12	14.1
Post-traumatic stress disorder	7	8.2
Complex post-traumatic stress disorder	1	1.2
**Bipolar spectrum disorders**	**5**	**5.9**
Bipolar I disorder	1	1.2
Bipolar II disorder	4	4.7
**Somatic symptom-related disorders**	**5**	**5.9**
**Attention-deficit/hyperactivity disorder**	**3**	**3.5**
**Obsessive-compulsive-related disorders**	**2**	**2.4**
**Others [a]**	**3**	**3.5**

(b) Comorbid diagnostic categories

**Comorbid diagnostic category [b]**	**n**	**%**
Trauma- and stressor-related disorders	16	18.8
Depressive disorders	13	15.3
Anxiety disorders	11	12.9
Somatic symptom-related disorders	3	3.5
Attention-deficit/hyperactivity disorder	3	3.5
Bipolar spectrum disorders	1	1.2
Obsessive-compulsive-related disorders	1	1.2

[a]Others comprise pathological gambling, an eating disorder and depersonalization/derealization disorder (one participant each).
[b]Categories are not mutually exclusive; a participant may contribute to more than one. Lower-ranked differential formulations qualified as less likely were not counted. Symptom-level descriptions or psychosocial circumstances, such as panic attacks or bereavement, were counted in the corresponding category. Forty-three participants (50.6%) had at least one comorbid diagnosis in the categories listed; one comorbid category was recorded in 38 participants and two in 5. An eating disorder and Cluster C personality traits were each recorded in one participant who also had a listed comorbid category and are not shown.

Table S5. Sensitivity analysis reincluding participants with non-calculable BMI.

The primary analysis used complete cases. Participants who completed the self-report instruments but whose body mass index could not be calculated because of missing anthropometric data were therefore excluded. To determine whether this exclusion influenced the findings, the analyses were repeated with these participants reincluded in two ways: with missing BMI replaced by the sample mean, and with BMI omitted from the covariate set. As a result, reinclusion did not alter the direction or significance of any principal association, so exclusion of these participants under complete-case analysis does not affect the conclusions. Because single mean substitution is known to underestimate variance, complete-case analysis was retained for the primary results, and the reinclusion analysis is reported here as a sensitivity check.

Path/statistic	Primary analysis	Reincluded, BMI mean-substituted	Reincluded, BMI omitted
Anger-out → cortisol			
β	-0.311	-0.310	-0.359
p	0.022	0.020	0.007
n	61	63	63
Anger-out → log cortisol/DHEA-S			
β	-0.278	-0.278	-0.319
p	0.037	0.034	0.015
n	59	61	61
Anger-in → log IL-6			
β	0.294	0.289	0.336
p	0.033	0.033	0.013
n	61	63	63
Social anxiety → log IL-6			
β	0.400	0.395	0.451
p	0.001	0.001	<0.001
n	63	65	65

β, standardized regression coefficient. Covariates were age, sex and body mass index, except in the rightmost column where body mass index was omitted. All four paths retained statistical significance under both approaches, and effect sizes increased when body mass index was omitted.

Table S6. Additional sensitivity analyses of the four associations that were nominally significant in the main models.

Model	n	B (95% CI)	*β*	*p*
Anger-out → cortisol				
Main model (age, sex, BMI)	61	−0.4027 (−0.7442, −0.0611)	−0.311	0.022
+ HbA1c	61	−0.3908 (−0.7388, −0.0427)	−0.302	0.028
+ current smoking	61	−0.4309 (−0.8009, −0.0610)	−0.333	0.023
Excluding primary bipolar spectrum diagnosis	56	−0.3838 (−0.7368, −0.0308)	−0.295	0.034
+ broad diagnostic category	61	−0.3522 (−0.6834, −0.0211)	−0.272	0.038
Anger-out → log cortisol/DHEA-S ratio				
Main model (age, sex, BMI)	59	−0.0382 (−0.0742, −0.0023)	−0.278	0.037
+ HbA1c	59	−0.0333 (−0.0686, 0.0019)	−0.242	0.064
+ current smoking	59	−0.0365 (−0.0753, 0.0023)	−0.265	0.065
Excluding primary bipolar spectrum diagnosis	54	−0.0381 (−0.0762, −0.0000)	−0.278	0.050
+ broad diagnostic category	59	−0.0321 (−0.0667, 0.0025)	−0.233	0.068
+ DHEA-S assay platform	59	−0.0383 (−0.0744, −0.0021)	−0.278	0.039
Samples measured before the platform change only	57	−0.0353 (−0.0707, 0.0001)	−0.265	0.051
Anger-in → log IL-6				
Main model (age, sex, BMI)	61	0.0370 (0.0031, 0.0709)	0.294	0.033

Model	n	B (95% CI)	β	p
+ lifetime psychotropic exposure	61	0.0405 (0.0057, 0.0752)	0.322	0.023
+ HbA1c	61	0.0360 (0.0021, 0.0698)	0.286	0.038
+ current smoking	61	0.0484 (0.0126, 0.0843)	0.385	0.009
Excluding primary bipolar spectrum diagnosis	56	0.0428 (0.0049, 0.0807)	0.324	0.028
+ broad diagnostic category	61	0.0390 (0.0033, 0.0748)	0.310	0.033
Social anxiety (LSAS total) → log IL-6				
Main model (age, sex, BMI)	63	0.0069 (0.0028, 0.0111)	0.400	0.001
+ HbA1c	63	0.0067 (0.0024, 0.0109)	0.383	0.003
+ current smoking	63	0.0084 (0.0042, 0.0127)	0.484	<0.001
Excluding primary bipolar spectrum diagnosis	58	0.0074 (0.0030, 0.0118)	0.420	0.001
+ broad diagnostic category	63	0.0072 (0.0028, 0.0115)	0.412	0.002

Linear regression models adjusted for age, sex and body mass index (BMI), with the additional covariate or sample restriction indicated. B, unstandardized coefficient; β, standardized coefficient; CI, confidence interval. Broad diagnostic category: depressive, anxiety, trauma- and stressor-related or other disorders, based on the primary diagnosis. Bipolar spectrum diagnoses: one bipolar I and four bipolar II disorders. DHEA-S was measured by enzyme immunoassay until September 2025 and by chemiluminescent immunoassay from 1 October 2025; two samples in the ratio analysis were measured after the change. Models adjusted for depressive symptom severity and psychotropic exposure for the anger-out and social anxiety associations are reported in Table 5 of the main text. These analyses are exploratory and were not adjusted for multiplicity.

Table S7. Left-censored Tobit models for log IL-6.

Model	n	B (95% CI)	β	p
Anger-in → log IL-6				
Tobit model (age, sex, BMI)	61	0.0396 (0.0070, 0.0721)	0.315	0.017
Tobit model + PHQ-9	61	0.0362 (−0.0026, 0.0749)	0.288	0.067
Social anxiety (LSAS total) → log IL-6				
Tobit model (age, sex, BMI)	63	0.0072 (0.0031, 0.0112)	0.413	<0.001
Tobit model + PHQ-9	63	0.0080 (0.0029, 0.0131)	0.461	0.002

IL-6 values below the limit of detection (1.50 pg/mL; n = 19) were treated as left-censored at log(1.50). Standardized coefficients for Tobit models were derived as B × SD(predictor) / SD(log IL-6). The conclusions were the same as in the main models, in which values below the limit of detection were replaced with LOD/√2 (Table 5 of the main text).
